# Medical News and Miscellany

**Published:** 1898-12

**Authors:** 


### Medical News and Miscellany.

**Now** is a good time to remit for subscription to the Red Back.

---

**Dr. Edward B. Jackson** has removed from Houston to San Antonio.

---

**Dr. M. Brandenburg** has removed from Galveston, Texas to Ardmore, I. T.

---

**The Austin District Medical Society** will meet in Austin December 21st.

---

**The** address of Dr. C. M. Ramsdell is changed from Lampasas, Texas to Cove, Oregon.

---

**A Long Arm:** "I don't like him;" she said, "he tried to put his arm around me four times in the parlor last night."

---

**Dr. Daniel,** for several years secretary of the Quarantine Department of Texas, resigned his position December. 1st.

**Hymeneal.**—The JOUREAL acknowledges receipt of cards announcing the marriage, to take place at Houston, Texas, Dec. 21 (inst.), of Dr. W. N. Murphy, of Austin, to Miss Ora G. Christian, daughter of Dr. Geo. W. Christian, of Houston.

---

**Wanted**—Agents for "History of the Spanish-American War," by Henry Watterson. A complete, authentic history; illustrated with over 76 full-page half-tones and many richly colored pictures. Large royal octavo volume, superb outfit, postpaid for only 50 cents (stamps taken). Most liberal terms given. The greatest opportunity of the year. Address: THE WERNER COMPANY, Akron, Ohio.

---

**Dr. Vard H. Hulen,** the distinguished oculist of Galveston, has removed to New York City and has offices at 71 W. 49th street. Dr. Hulen in renewing his subscription to the JOURNAL, writes: "I did not like to leave Texas, but wished for a larger field for my work. I want the 'Red Back' to follow me."

We commend Dr. Hulen to the kindly regard and esteem of the profession in his new home, as a gentleman and scholar worthy of the "right hand of fellowship."

---

**Asylum Appointments Under the New Regime:** Dr. B. M. Worsham, the present incumbent, will be reappointed Superintendent of the Lunatic Asylum at Austin. Dr. M. L. Graves of Waco will succeed Dr. McGregor as Superintendent of the State Lunatic Asylum at San Antonio, and Dr. J. T. Wilson of Sherman will be Superintendent of the one at Terrell. Dr. E. P. Becton was reappointed Superintendent of the Blind Institute at Austin. On authority of Governor-elect Sayers. No quarantine appointments have been announced yet.

---

**The Quarantine Convention Called in Memphis** last month resulted in the recommendation to Congress of the passage of a law creating a Bureau of Health in the Treasury Department. The proposed bureau would frame all regulations necessary for the enforcement of all national sanitary laws. It would have charge, consequently of marine sanitation, interstate quarantines and other preventive measures during epidemics as well as the decision concerning the time when such quarantines are necessary. The recommendation contemplates also the creation of an Advisory Board, composed of one representative from each State of the Union. All regulations of the bureau would be submitted to the latter board, which would have the power to approve such regulations or not, the regulations to become effective only after their approval; the board would act upon a majority vote.—*N. O. Med. Journal.*

---

**Obituary.**—Died at his home in Brazos county, near Bryan, Texas, September 8, 1898, Dr. J. Marion Soles, aged 64. At the semi-annual meeting of the Brazos Valley Medieal Association held at Rockdale, Texas, November 15-16, 1898, Dr. B. F. Watkins read a paper on the life and service of Dr. Soles, in which he paid a high tribute to his character and attainments as a physician. We extract from Dr. Watkins' paper the following biographical data:

"Dr. Soles was born in Lowndes Co. Ala., December 18, 1834, graduated M. D. from the South Carolina Medical College at Charleston in 1856, and was practicing medicine in Alabama when the war of Secession came on. He volunteered as a private soldier in the Southern army. He served in the ranks three years, and was then made assistant surgeon, in which capacity he served the Confederacy till the close of the war. He came to Texas after the war (1867), settling in Grimes county, and afterwards removed to Brazos, where he resided until the date of his death."

The association adopted appropriate resolutions on the death of Dr. Soles.

